# Orm proteins control ceramide synthesis and endocytosis via LCB-mediated Ypk1 regulation

**DOI:** 10.1016/j.jlr.2024.100683

**Published:** 2024-10-28

**Authors:** Jihui Ren, Robert Rieger, Nivea Pereira de Sa, Douglas Kelapire, Maurizio Del Poeta, Yusuf A. Hannun

**Affiliations:** 1 Department of Medicine, Stony Brook University, Stony Brook, NY; 2 Stony Brook Cancer Center, Stony Brook University, Stony Brook, NY; 3 Biological Mass Spectrometry Core Facility, Stony Brook University, Stony Brook, NY; 4 Department of Microbiology and Immunology, Stony Brook University, Stony Brook, NY; 5 Northport Veterans Affairs Medical Center, Northport, NY

**Keywords:** Orm1/2, SPT, LCBs, Ypk1, endocytosis, sphingolipids, lipids, lipidomics, cell signaling, enzymology/enzyme regulation

## Abstract

Sphingolipids (SPLs) are major components of cell membranes with significant functions. Their production is a highly-regulated multi-step process with the formation of two major intermediates, long chain bases (LCBs) and ceramides. Homologous Orm proteins in both yeast and mammals negatively regulate LCB production by inhibiting serine palmitoyltransferase (SPT), the first enzyme in SPL de novo synthesis. Orm proteins are therefore regarded as major regulators of SPL production. Combining targeted lipidomic profiling with phenotypic analysis of yeast mutants with both ORM1 and ORM2 deleted (*orm1/2Δ*), we report here that Ypk1, an AGC family protein kinase, signaling is compromised in an LCB-dependent manner. In *orm1/2Δ*, phosphorylation of Ypk1 at its activation sites is reduced, and so is its in vivo activity shown by reduced phosphorylation of Ypk1 substrate, Lac1, the catalytic component of ceramide synthase (CerS). A corresponding defect in ceramide synthesis was detected, preventing the extra LCBs generated in *orm1/2Δ* from fully converting into downstream SPL products. The results suggest that Orm proteins play a complex role in regulating SPL production in yeast *S. cerevisiae* by exerting an extra and opposite effect on CerS. Functionally, we define endocytosis and an actin polarization defect of *orm1/2Δ* and demonstrate the roles of Ypk1 in mediating the effects of Orm proteins on endocytosis. Collectively, the results reveal a previously unrecognized role of yeast Orm proteins in controlling ceramide synthesis and their function in endocytosis through regulating Ypk1 signaling.

Sphingolipids (SPLs) are major components of the cell membranes of all eukaryotes with significant biological activities (1). The de novo synthesis of SPLs is a multi-step process with the formation of two major groups of intermediate products, long chain bases (LCBs) and ceramides (Fig. 1). Serine palmitoyltransferase (SPT) catalyzes the rate-limiting step of condensation between serine and palmitoyl-CoA to produce LCBs (2, 3). Other amino acids and fatty acids can also be utilized by SPT for the production of non-canonical LCBs (4, 5, 6). LCBs are then N-acylated with another fatty acid by ceramide synthases (CerSs) to form ceramides (7, 8). The hydroxy group at the C1 position of ceramides can then be linked with various moieties, resulting in the formation of complex SPLs (9, 10, 11). The pathway also operates in reverse to convert complex SPLs to ceramides and LCBs by various hydrolases (12, 13, 14). LCBs can be phosphorylated by sphingosine kinases to form LCB-1-Ps (15) which are subject to degradation by a lyase in yeast called Dpl1 and this is the only known exit point for the SPL pathway (16, 17).

**Fig. 1:**
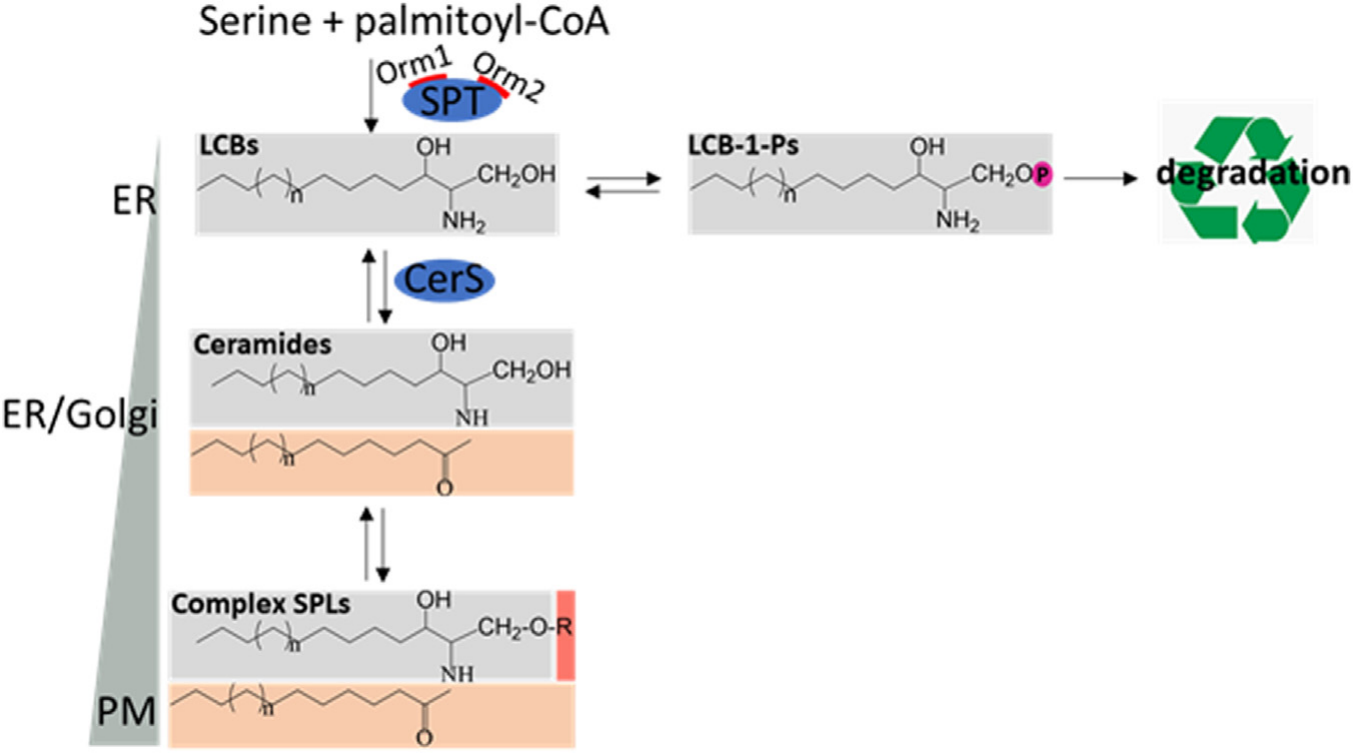
Schematic diagram of sphingolipid metabolic pathway in yeast *S. cerevisiae*. The chemical structures of complex SPLs and the major intermediates, including LCBs, ceramides and LCB-1-Ps, are shown. Sphingosine backbone, acyl chain of ceramides, and a head group of complex SPLs are labeled in gray, orange, and red, respectively. P in purple circle marks the phosphate group. Also shown are their cellular distribution and relative abundance. Blue ovals indicate enzymes required for the synthesis of LCBs and ceramides, respectively.

Complex SPLs are major components of the plasma membrane (PM) and are hypothesized to form special microdomains called lipid rafts, which have been implicated to function in immune signaling and host-pathogen interactions (18, 19). Specific complex SPLs such as sphingomyelins and glycosphingolipids are important for diverse physiological processes such as the regulation of transmembrane signaling (20, 21). Glucosylceramides in pathogenic fungi play key roles in their virulence (22, 23). Other than acting as intermediates in the SPL biosynthesis pathway, LCBs, LCB-1-Ps, and ceramides are also bioactive molecules with their own function. In vertebrates, sphingosine-1-phopshate (S1P) executes its function in angiogenesis and immune cell migration through binding to G-protein coupled S1P receptors (24). Ceramides are produced in response to various extracellular stimuli to control stress responses by regulating cell cycle and apoptosis through activating protein phosphatases (25). Sphingosine, the major LCB in mammalian cells, is a potent inhibitor of protein kinase C (PKC) (26, 27). LCBs in yeast are proposed to be a major mediator of heat stress response (28). Yet compared with LCB-1-Ps and ceramides, the role of LCBs as bioactive signaling molecules is less clear due to a lack of knowledge of upstream regulators that control their production and downstream effectors that execute their function.

SPL metabolism is highly regulated. In yeast, the SPT complex (SPOT) is composed of catalytic subunits Lcb1/Lcb2 as well as regulatory subunits, including Tsc3, phosphatidylinositol 4 -phosphate (PI4P) phosphatase Sac1, and Orm1/2, the latter acting to suppress SPT activity (29, 30, 31, 32, 33). SPT structure and function are highly conserved from yeast to humans, where *ORMDL1/2/3* are the orthologs of Orm1/2, sharing the same physical interactions and negative regulatory effects on SPT (4, 29, 34, 35, 36, 37, 38). SPT is activated when Orm proteins are dissociated from the SPOT complex. When Orm proteins in yeast are phosphorylated or removed together, SPT is activated leading to increased LCB production. Since SPT catalyzes the rate-limiting step of SPL biosynthesis, the increased LCBs are presumed to be able to flux through the pathway leading to the production of more complex SPLs. Hence, Orm proteins are regarded as central regulators of SPL production.

Using mass spectrometry-based methods to monitor SPL flux, we here find that the induced LCBs caused by ORM1/2 knockout (*orm1/2Δ*) are not capable of fully fluxing through the pathway with a defect in their conversion into ceramides. The impaired ceramide production in *orm1/2Δ* is caused by inactivation of the AGC family protein kinase Ypk1, which phosphorylates and activates CerS catalytic subunits Lac1/Lag1. We then show Ypk1 activity is controlled by Orm proteins in an LCB-dependent manner. Other than phosphorylating Lac1 and regulating SPL metabolism, Ypk1 and its close homolog Ypk2 also play important roles in a plethora of cellular processes, such as endocytosis, autophagy, vacuole acidification, ROS production, mitochondria respiration, osmolarity maintenance, genome stability, and the calcineurin-mediated stress response (39, 40, 41, 42, 43). Here we demonstrate that Ypk1 also mediates Orm1/2’s effects on endocytosis by defining a severe endocytosis defect that can be rescued by reconstituting Ypk1 activity in *orm1/2Δ*. Taken together, our results reveal novel functions of Orm proteins in ceramide synthesis and endocytosis through regulation of LCB production and Ypk1 activity. This discovery suggests the potential role of Orm proteins as upstream regulators to control Ypk1-mediated biological functions via regulating LCB production.

## Materials and methods

### Yeast strains and growth conditions

All *S. Cerevisiae* strains used in this study are listed in Table 1. Yeast was grown at 30°C in either liquid YPD (yeast extract peptone dextrose) or synthetic defined (SD) medium. Yeast knockout mutants were generated via homologous recombination with specific PCR products to replace the gene of interest with antibiotic or auxotrophic markers (44). Modification of genes of interest at the genomic locus (introducing point mutations or adding protein tags) was achieved using a CRISPR system. The haploid yeast strain with a gene of interest knocked out and replaced with KanMX was transformed with a plasmid encoding guide RNA targeting the KanMX locus and coding sequence for Cas9 (pRS425-Cas9-kanMx), and PCR products containing the modified gene with flanking regions homologous to ∼200 bp up- and downstream of gene of interest. The surviving colonies were confirmed to harbor the gene of interest via sequencing.

**Table 1:** *S. Cerevisiae* strains used in this study

Strain	Genotype	Source/reference
BY4741	MATa his3Δ1 leu2Δ0 met15Δ0 ura3Δ0	Dharmacon™
*cka2Δ*	BY4741 *cka2Δ*::KanMX	Dharmacon™
*lcb4Δ*	BY4741 *lcb4Δ*::KanMX	Dharmacon™
*orm1/2Δ*	BY4741 *orm1*Δ::KanMX *orm2Δ*::URA3	This study
*orm1/2Δtsc3Δ*	BY4741 *orm1Δ*::KanMX *orm2Δ*::URA3 *tsc3Δ::*LEU2	This study
*orm1/2ΔYpk2*^*D239A*^	BY4741 *orm1Δ*::KanMX *orm2Δ*::URA3 Ypk2^D239A^	This study
*Flag-Lac1*	BY4741 3XFlag-Lac1	This study
*Flag-Lac1*^*S23AS24A*^	BY4741 3XFlag-Lac1^S23AS24A^	This study
*orm1/2Δ Flag-Lac1*	BY4741 *orm1Δ*::KanMX *orm2Δ*::URA3 Flag-lac1	This study
*orm1/2Δ tsc3Δ Flag-Lac1*	BY4741 *orm1Δ*::KanMX *orm2Δ*::URA3 *tsc3Δ*::LEU2 Flag-lac1	This study
*orm1/2Δ Ypk2*^*D239A*^*Flag-Lac1*	BY4741 *orm1Δ*::KanMX *orm2Δ*::URA3 Flag-lac1 Ypk2^D239A^	This study
Ste2-GFP	BY4741 Ste2-GFP::His	Dharmacon™
*orm1/2Δ*Ste2-GFP	BY4741 Ste2-GFP::His *orm1Δ*::KanMX *orm2Δ*::URA3	This study

### Lipid analysis of LCBs and ceramides

Lipid extraction and quantification of LCBs and ceramides via HPLC-ESI-MS/MS was performed as previously described with minor modifications (5, 45). Briefly, yeast cells cultured in SD (synthetic defined) medium were collected via centrifugation. C17-Sphingosine or C18:1 ceramide (Avanti® Polar Lipids, Inc), both at 50 pmol, were added as internal standards for LCBs and ceramides, respectively. Lipids were extracted, dried, and resuspended in 200 μl mobile phase B solution (0.2% formic acid and 1 mM ammonium formate in methanol). Aliquots of 10 μl were injected into an HPLC system linked to a mass spectrometer ESI source, which was operated in multiple reaction monitoring (MRM) positive ionization mode, with the following transition parameters: C17-sphingosine (286.2/268.2); C18:1Cer (564.5/264.3); 3KDS (300.2/270.3); DHS (302.3/60.1); PHS (318.3/300.3); PHS-1-P (398.2/300.3); C26-PHC (696.4/282.3); C26-PHC-OH (712.7/282.2); C17-PHS (304.3/286.3); C17-PHS-1P (384.3/268.3); C26-PHC (d17) (682.5/268.2). Data were collected and processed using Xcalibur software. The relative abundance of each SPL species was calculated by the peak area of the compound normalized to the internal standard signal and cell number. The peak area of each compound detected was within the linear range of the calibration curve.

### Lipid analysis of complex SPLs

Yeast was cultured in SD medium with 2% glucose to logarithmic phase.∼ 0.2–2 × 10^8^ cells were collected and subjected to lipid extraction as described previously (45). C26-Cer-IPC (44:1:2) at 50 pmol was added to each sample as an internal standard. The dried samples were resuspended in 150 μl of 1 mM ammonium acetate in 99% methanol and separated on a Thermo Accela HPLC system. Samples were injected (10 μl) onto a Peeke Scientific Spectra C8 HPLC column (150 × 3 mm). Injected lipids were eluted with a linear gradient starting with 70% B (1 mM ammonium acetate in 99% methanol) and 30% A (1 mM ammonium acetate in 10% methanol), increased to 90% B and 10% A, with the flow at 500 μl/min. The HPLC was coupled to the ESI source of a Thermo TSQ Quantum Ultra triple quadrupole mass spectrometer. The mass spectrometer was operated in the negative ion mode with the high voltage set to −3.1 kV, vaporizer temperature at 350°C, sheath gas pressure set to 60 mTorr, auxiliary gas pressure at 15 mTorr, and a capillary temperature of 350°C. The tube lens was set to 190. The collision cell was operated at 1.5 mTorr of argon. Transitions for each IPC were monitored at 100 ms dwell time during the experiment. The following transitioning parameters were used: C26-Cer-IPC (918.5/240.9); C26-PHC-IPC (936.5/240.9); C26-PHC-MIPC (1,098.5/421.1); C26-PHC-M(IP)_2_C (669.9/240.9); C26-PHC-OH-IPC (952.6/240.9); C26-PHC-OH-MIPC (1,114.5/421.1); C26-PHC-OH-M(IP)_2_C (677.9/240.9). Processing of the collected data was performed using Xcalibur 2.2 software. Data were normalized to the internal standard and cell number.

### Measurement of deuterated serine flux into SPL pathway

Yeast was cultured in SD medium with 2% glucose until cell density reached between 0.2–2 × 10^7^ cells/ml. 7.6 mM L-serine (3,3)-D2 (Cambridge Isotope Laboratories, Inc) was added to the culture. ∼10 ml of yeast was collected at 0-, 15-, 30-, 60-, 90- and 120-min timepoints. Samples were kept on ice with 5% trichloroacetic acid for 10 min before centrifugation at 3000 rpm for 5 min. Lipids were extracted and subjected to HPLC-ESI-MS/MS analysis as described in “Lipid analysis of LCBs and ceramides”. The following transitioning parameters were used: D2-3KDS (302.2/270.3); D2-DHS (304.3/62.1); D2-PHS (320.3/302.3); D2-PHS-1-P (400.2/302.3); D2-C26-PHC (698.2/680.2). The relative abundance of each SPL species was calculated as described in “Lipid analysis of LCBs and ceramides”, except that background signals from corresponding endogenous compounds containing two C13 isotopes were deducted.

### Western blot

Whole-cell extracts were generated via protein precipitation with 5% trichloroacetic acid, followed by bead-beating. Protein precipitates were washed with acetone twice before dissolving in 2XLSB (laemmli sample buffer). Proteins were resolved on SDS-PAGE (10% acrylamide with or without 40 μM Phos-Tag (FUJIFILM Wako chemicals) and 80 μM MnCl_2_) at 100v and transferred to a nitrocellulose membrane. Membranes were probed with an ANTI-FLAG M2 antibody (1:5,000, Cat #F3165, MilliporeSigma), α-alpha Tubulin (YOL 1/34) (1:2,500, Cat# NB100-1639, Novus Biologicals), α-PKC (pan)zeta T410 (190D10) (1:1,000, Cat20605, Cell Signaling Technology) for phospho-Ypk1(T504) detection, Rabbit polyclonal α-Ypk1 from Dr Mitsuaki Tabuchi’s group (1:1,000) (46) and a rabbit polyclonal α-phospho-Ypk1(T662) from Dr Ted Powers’ group (1:20,000) (47). Membranes were then incubated with secondary antibodies against mouse, rabbit, or rat IgG conjugated with horseradish peroxidase (1:5,000, Jackson ImmunoResearch). Protein bands were visualized using Pierce™ ECL western blotting substrates (ThermoFisher) on the chemiDoc Imaging System (Bio-Rad).

### Endocytosis assays

Fluid-phase endocytosis was measured based on the uptake of Lucifer Yellow CH, Lithium salt (Invitrogen) as previously described (48). Plasma membrane endocytosis was assayed using FM4-64 (49). Briefly, yeast cells were cultured in YPD to logarithmic phase, collected, and incubated in 100 μl YPD at 10^8^ cells/ml with 4 mg/ml Lucifer Yellow or 16 μM FM4-64 at 30°C for 30 min. Cells were washed with cold PBS/10 mM sodium azide/10 mM sodium fluoride before mounting on slides for observation under an Olympus BX53 fluorescence microscope (Olympus Corporation of the Americas, Center Valley, PA) with excitation/emission wavelengths at 428/536 for LY and 505/725 for FM4-64.

### Actin staining

Rhodamine-phalloidin staining of yeast actin patches/cables was performed as described previously (50). Briefly, yeast cells were cultured in YPD to the logarithmic phase, fixed with 4% formaldehyde, permeabilized with 0.2% Triton X-100, and incubated with 3.3 μM Rhodamine phalloidin for 1 h at 4°C. After thorough washing with PBS, cells were mounted on slides for observation under a fluorescence microscope equipped with a rhodamine filter.

## Results

### Deletion of ORM1/2 has different effects on the cellular level of LCBs versus ceramides and complex SPLs

**Fig. 2:**
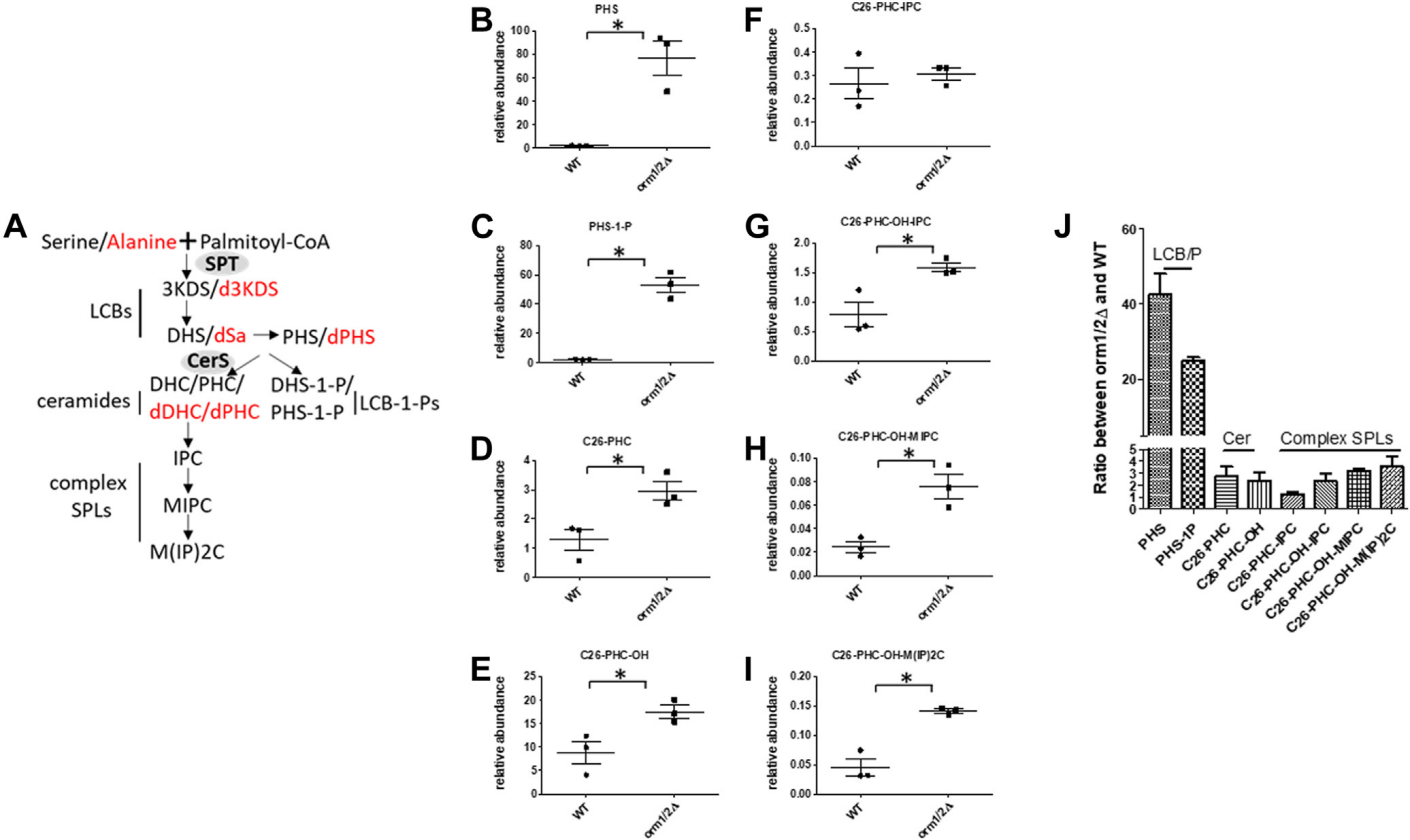
Deletion of ORM1/2 has different effect on cellular level of LCBs versus ceramides and complex SPLs. A: A simplified diagram of de novo synthesis pathway of sphingolipids in *S. cerevisiae*. The major groups of SPL species measured in this study are shown. SPT and CerS are highlighted. Deoxy-SPLs derived from alanine are in red. B–I: SPL profile of WT versus *orm1/2Δ*. Yeast cells were cultured in YPD medium to exponential growth phase, whereafter lipids were extracted and quantified via HPLC-ESI-MS/MS. J: Fold changes in indicated SPL species in *orm1/2Δ* versus WT. The data are shown as mean with SEM and were analyzed using an unpaired two-tailed *t* test in GraphPad Prism5. ∗*P* < 0.05. 3KDS, 3-ketodihydrosphingosine; C26-PHC, C26-phytoceramide; C26-PHC-OH, hydroxy C26-phytoceramide; C26-PHC-OH-IPC, inositol phosphoryl C26-PHC-OH; C26-PHC-OH-MIPC, mannose-inositol-phosphoryl C26-PHC-OH; C26-PHC-OH-M(IP)2C, mannose-(inositol-P)_2_-C26-PHC-OH; DHC, dihydroceramide; DHS, dihydrosphingosine; DHS-1-P, dihydrophingosine-1-phosphate; IPC, inositol phosphorylceramide; MIPC, mannosylinositol phosphorylceramide; M(IP)2C, mannosyldiinositol phosphorylceramide; PHS, phytosphingosine; PHC, phytoceramide; PHS-1-P, phytosphingosine-1-phosphate.

Yeast *ORM1/2* deletion mutants (*orm1/2Δ*) exhibit a dramatic increase in LCB levels, yet controversy exists regarding their SPL profiles at the level of products downstream of LCBs, including ceramides and complex SPLs. While increases in SPLs have been reported at all levels (29), others observed decreased ceramide (31) and complex SPLs (51) in *orm1/2Δ* mutants. This controversy has led to speculation that Orm1/2 has roles beyond acting as negative regulators of SPT. Here, we developed high-performance liquid chromatography-electrospray ionization-tandem mass spectrometry (HPLC-ESI-MS/MS) methods to quantitate the levels of targeted SPLs and intermediate species, including LCBs, ceramides, and complex SPLs in WT versus *orm1/2Δ* strains. The de novo synthesis pathway of yeast SPLs and the major intermediates are illustrated in Fig. 2A. Although Serine is the predominant substrate, other amino acids can also be utilized by SPT to form non-canonical SPLs. Under certain culture conditions, there is a measurable amount of alanine-derived deoxy-SPLs in yeast (5). We first measured the serine-derived SPL profile of WT versus *orm1/2Δ*. The results show an increase in all the SPL species in *orm1/2Δ* including LCB (Fig. 2B), LCB-phosphate (Fig. 2C), ceramide (“ceramides” in this study is used to refer generically to yeast dihydroceramides and phytoceramides) (Fig. 2D–E), and complex SPL levels (Fig. 2F–I). Interestingly, the accumulation of LCBs (∼40×) and LCB-phosphate (∼20×) was much greater than that of ceramide (∼2–4×) and complex SPLs (∼2–4×) (Fig. 2J). This could simply indicate that increased LCB production caused by removing Orm1/2 from SPT complex exceeds the capacity of downstream CerS. There is also a possibility that the accumulation of LCBs/LCB-1-Ps results from inhibition of CerS activity in addition to increased SPT activity.

### *orm1/2Δ* mutants are defective in ceramide production

**Fig. 3:**
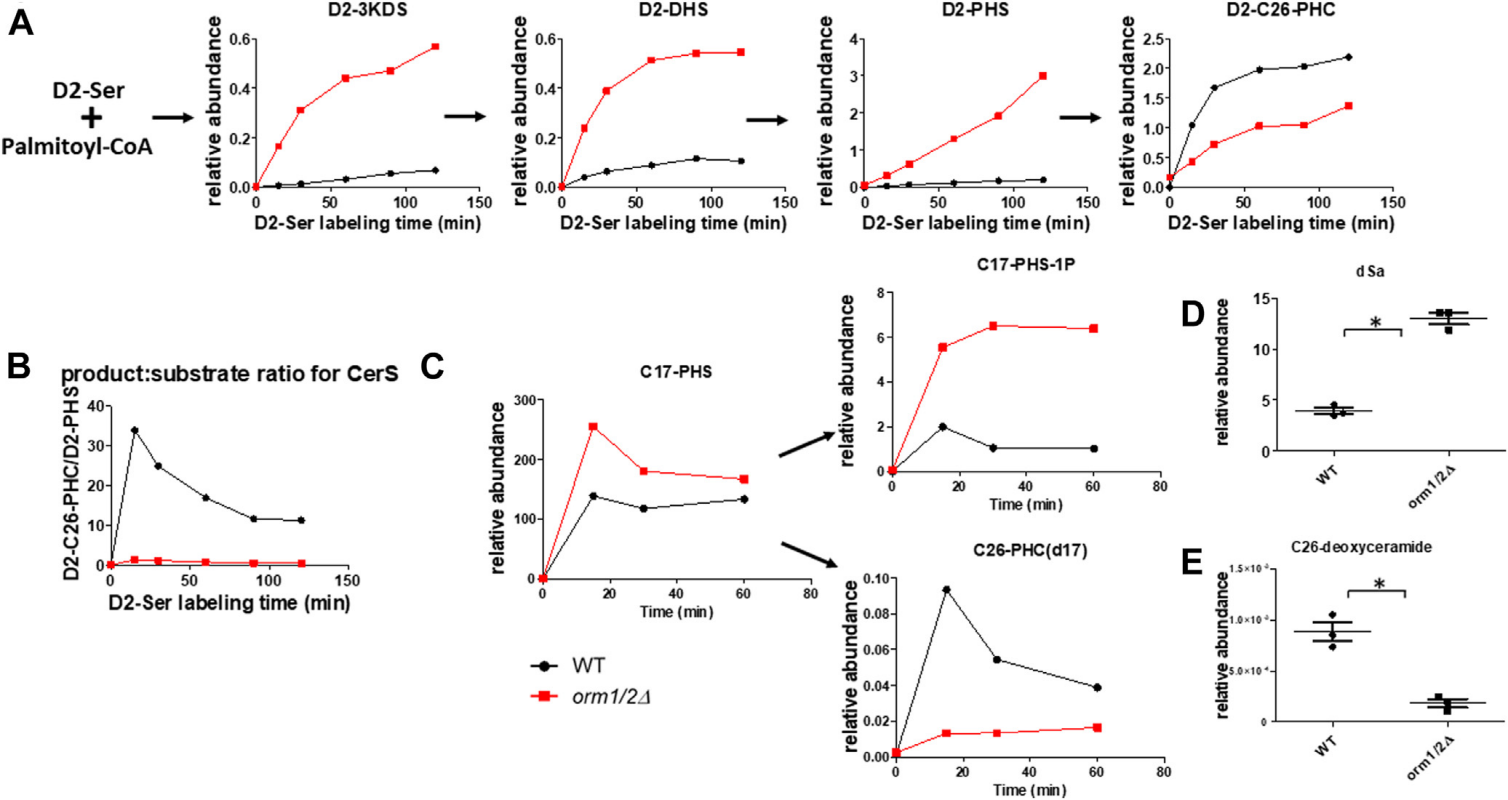
*orm1/2Δ* mutants are defective in ceramide production. A: Incorporation of L-Ser (3,3)-D2 into deuterated (D2-) LCBs and ceramides. B: The ratio between D2-C26-PHC and D2-PHS was plotted at each time point to reflect CerS activity. C: Conversion of C17-PHS into C17-PHS-1P and C26-PHC(d17). 7.6 mM D2-Ser (A) or 10 μM C17-PHS (C) were added to the yeast culture. Yeast cells were collected at indicated time points before being subjected to lipid extraction and quantification by HPLC-ESI-MS/MS. The data shown are representative results of three independent experiments. D, E: Deoxy-SPL profile of WT versus *orm1/2Δ*. Yeast cells were cultured in a synthetic defined growth medium to exponential growth phase before being subject to lipid extraction and quantification via HPLC-ESI-MS/MS. dSa, deoxy-sphinganine.

Since the steady-state measurement of bulk SPL levels could reflect a possible blockage of ceramide production in *orm1/2Δ* mutants, we sought to evaluate SPL flux in WT and *orm1/2Δ* yeast. We first monitored the incorporation of stable isotope-labeled serine (L-Ser (3,3)-D2, which we call D2-Ser for short) into the de novo pathway of SPL synthesis (Fig. 3A) (5, 45). This allowed us to follow the incorporation of D2-Ser into D2-3KDS, the immediate product of SPT, and then the reduction of D2-3KDS into D2-DHS, followed by its hydroxylation into D2-PHS. We further followed D2-PHS conversion into ceramide via acylation with hexacosanoic acid (C26:0) to form D2-C26-PHC. The *orm1/2Δ* mutant showed a marked increase in LCB synthesis. Yet not only was there no additional D2-C26-PHC produced from the extra D2-PHS generated in *orm1/2Δ* yeast, but it was also produced at a much lower level than in the WT strain, showing a clear defect in ceramide production. This defect in ceramide production in the double knockout was also assessed by the product: substrate ratio which was dramatically lower than in the WT (Fig. 3B). In order to further test the ceramide synthesis defect in *orm1/2Δ*, we added C17-PHS to the yeast culture and measured its conversion into ceramide (C26-PHC(d17)) directly. Figure 3C shows decreased formation of C17-PHS derived ceramides, with the accumulation of C17-PHS and C17-PHS-1-P. Together, these results demonstrate reduced in vivo activity of ceramide synthase in *orm1/2Δ*. Finally, we measured the level of deoxy-ceramide, which cannot be converted into downstream complex SPLs due to the lack of hydroxy group at the C1 position of SPL backbone, and whose steady-state level is a more direct reflection of CerS activity. We observed an increase in deoxy-sphinganine (dSa) level (Fig. 3D) and a decrease in C26-deoxyceramide level (Fig. 3E). The results further support that CerS activity is impaired in *orm1/2Δ*. This extra ceramide synthesis defect caused by deleting both ORM1 and ORM2 explains the decreased accumulation of ceramides and complex SPLs compared with an accumulation of LCBs shown by the steady-state measurement.

### Decreased ceramide synthesis in *orm1/2Δ* mutants is caused by PHS-mediated Ypk1 inactivation

#### LCB accumulation causes impaired phosphorylation of Lac1 in the orm1/2Δ mutants

**Fig. 4:**
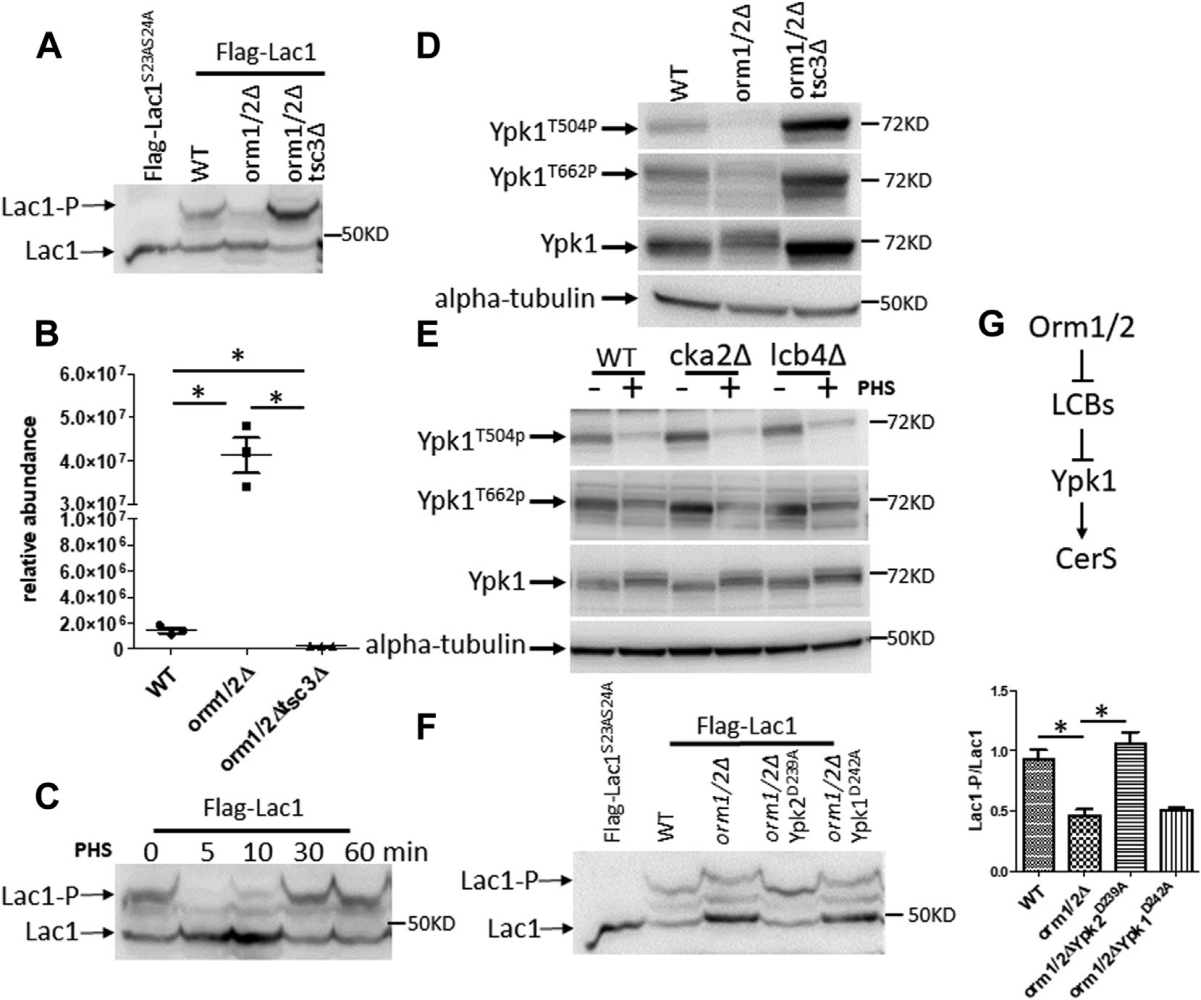
Orm proteins regulate CerS activity through PHS-modulated Ypk1 activation. A: Lac1 phosphorylation is decreased in *orm1/2Δ* yeast and can be corrected by deleting TSC3. Whole-cell extracts of the indicated strains containing Flag-Lac1 were resolved by phos-tag SDS-PAGE and immunoblotted with an anti-Flag antibody. B: LCB accumulation in *orm1/2Δ* yeast is abolished by deleting TSC3. Lipids were extracted and PHS level was measured with HPLC-ESI/MS/MS. C: Exogenously added PHS reduces Lac1 phosphorylation. WT yeast containing Flag-Lac1 were cultured to exponential phase and treated with 10 μM PHS at the indicated time points. D: Ypk1 phosphorylation is decreased in *orm1/2Δ* and can be corrected by deleting TSC3. Whole-cell extracts of the indicated strains were resolved by SDS-PAGE and immunoblotted with the indicated antibodies. E: Exogenously added PHS reduces Ypk1 phosphorylation. Indicated strains were cultured to exponential phase and treated with or without 10 μM PHS treatment for 5 min. F: Ypk2^D239A^ rescues the Lac1 phosphorylation defect in *orm1/2Δ* yeast. Whole-cell extracts of the indicated strains containing Flag-Lac1 were resolved by phos-tag SDS-PAGE and immunoblotted with an anti-Flag antibody. Band intensity measured using image J were used to calculate ratios between Lac1-P and Lac1 and are shown on the right. G: A diagram illustrating the relationships deduced from these results between Orm1/2/LCBs and Ypk1 on CerS. Western Blot shown here are representative of three biological replicates. ∗*P* < 0.05.

As part of the SPOT complex, Orm1/2 are well-established negative regulators of SPT, yet the lipid profiling revealed a surprising defect of ceramide production in *orm1/2Δ* strains. Here, we first investigated how CerS activity is compromised in *orm1/2Δ*. Yeast CerS activity is regulated via phosphorylation of its catalytic subunit Lac1^S23S24^ and its paralogue Lag1^S23S24^ by the AGC family kinase Ypk1 (52). In order to evaluate the phosphorylation status of Lac1^S23S24^, we generated WT and *orm1/2Δ* strains with 3XFLAG integrated into the N-terminus of the LAC1 genomic locus. Phosphorylated Lac1(Lac1-P) and its unphosphorylated counterpart (Lac1) in the yeast protein extract were separated through phosphate-affinity SDS-PAGE (also called phos-tag gels), with the phosphorylated form as the slower migrating band (53). A yeast strain expressing 3XFLAG-tagged Lac1^S23AS24A^ was also included as a phosphorylation-deficient control. Figure 4A shows that Lac1 phosphorylation was indeed greatly reduced in *orm1/2Δ*, with a much lower ratio between phosphorylated over unphosphorylated Lac1. This explains the decreased CerS activity determined through SPL flux measurements.

In order to determine if the increases in LCB production are responsible for the decreased phosphorylation of Lac1, we used a combination of genetic and pharmacologic manipulations. We first deleted TSC3, which encodes a small regulatory subunit of the SPT complex, and the deletion of TSC3 in WT cells results in reduced LCB levels (5). The defect of Lac1 phosphorylation in *orm1/2Δ* was not only rescued by deleting TSC3 in *orm1/2Δ*, but phosphorylation became the dominant form in *tsc3Δorm1/2Δ* (Fig. 4A)*.* Indeed, SPL analysis showed that TSC3 deletion eliminated the accumulation of LCBs (DHS and PHS) in *orm1/2Δ*, bringing their levels even lower than those in the WT (Fig. 4B). These results demonstrate a negative correlation between CerS activation and cellular LCB level. Next, PHS was added exogenously to WT yeast to evaluate its effect on Lac1-P. PHS was used instead of DHS because PHS is the most dominant LCB species in yeast. Figure 4C shows that 10 μM PHS acutely and potently inhibited Lac1-P. This effect was also transient, starting to fade at 10 min and completely disappearing after 30 min of treatment. This potent, yet transient, effect of PHS on Lac1-P strongly suggests PHS, rather than the downstream metabolites that it is converted into such as PHS-1-P and ceramides, exerts the inhibitory effect since the conversion occurs over a 10–30 min time frame (5).

#### LCB accumulation causes Ypk1 inactivation in orm1/2Δ mutants

Since Ypk1 is known to phosphorylate and activate Lac1/Lag1 in yeast, we sought to determine whether the reduction of Lac1-P in *orm1/2Δ* is due to lower Ypk1 kinase activity. Ypk1 activation requires its phosphorylation at T504, residing at the activation loop within the catalytic domain, by Pkh kinases. Activation also requires phosphorylation at S644/T662 within the C-terminal regulatory domain by the Torc2 complex (54, 55, 56). Using antibodies that specifically detect Ypk1^T504P^ or Ypk1^T662P^ for Western blot analysis, a ∼50% reduction for Ypk1^T504P^ and a ∼30% reduction of Ypk1^S644T662P^ signal were observed in the *orm1/2Δ* strain (Fig. 4D). The reduced Ypk1 phosphorylation also corresponded to changes in the migration of total Ypk1 detected in *orm1/2Δ*, where it migrated slower and split into two bands. We also noticed a minor yet consistent reduction in Ypk1 total protein level.

Next, it became important to determine the role of SPLs in the Ypk1 inhibition observed in *orm1/2Δ*. To this end, Ypk1 phosphorylation status was evaluated in *tsc3Δorm1/2Δ* triple mutants. The phosphorylation of Ypk1 at both T504 and T662 was not only recovered but was also greater than in the WT, at 200% and 120%, respectively upon deletion of TSC3 (Fig. 4D). We also observed a ∼2-fold increase in the level of total Ypk1 in *orm1/2Δtsc3Δ* compared with the WT, suggesting a possible role of Ypk1-P in Ypk1 stabilization. Importantly, these results point to a key role for SPLs in mediating the inhibition of Ypk1 in *orm1/2Δ.* To evaluate the effect of PHS on Ypk1 activation, exogenous PHS at 10 μM was added to WT yeast. Figure 4E shows that 5-min treatment exerted a potent inhibitory effect on both Ypk1^T504P^ and Ypk1^T662P^. To further determine the responsible SPLs (LCBs, their phosphates, or ceramides/complex SPLs), we evaluated the effects of PHS on Ypk1 activation status in yeast mutants cka2Δ and lcb4Δ, which exhibit reduced synthesis of ceramide and PHS-1-P, respectively (57, 58). Interestingly, the results showed that PHS had the same, if not a more pronounced effect on inducing Ypk1 dephosphorylation in these two strains, which strongly suggests that PHS, and not its downstream SPL metabolites, exerts the inhibitory effect on Ypk1.

#### CerS defect in orm1/2Δ mutants is caused by Ypk1 inactivation

The above data show that Ypk1 and CerS are both inactivated in *orm1/2Δ* as a consequence of LCB accumulation. In order to evaluate directly if the decreased Lac1-P is caused by Ypk1 inactivation, we tested whether reconstituting Ypk1 activity could rescue the phosphorylation defect of Lac1 in *orm1/2Δ*. The mutant allele of Ypk1 (Ypk1^D242A^) and its homologue Ypk2 (Ypk2^D239A^) have both been reported as constitutively active yet only Ypk2^D239A^ was identified in a multicopy suppressor screen that could rescue the growth of *tor2Δ*, yeast mutant without the essential TOR2 gene that encodes the major upstream regulator of Ypk1/2 (55, 59, 60). Replacing the WT locus of YPK2 with Ypk2^D239A^ in *orm1/2Δ* indeed reversed the level of Lac1-P from less than to more than that in the WT (Fig. 4F). Yet Ypk1^D242A^ showed little effect in rescuing Lac1-P defect of *orm1/2Δ* yeast. In summary, these data demonstrate that decreased ceramide synthesis in *orm1/2Δ* is caused by PHS-modulated Ypk1 inactivation (Fig. 4G).

### The *orm1/2Δ* strain displays an endocytosis defect

**Fig. 5:**
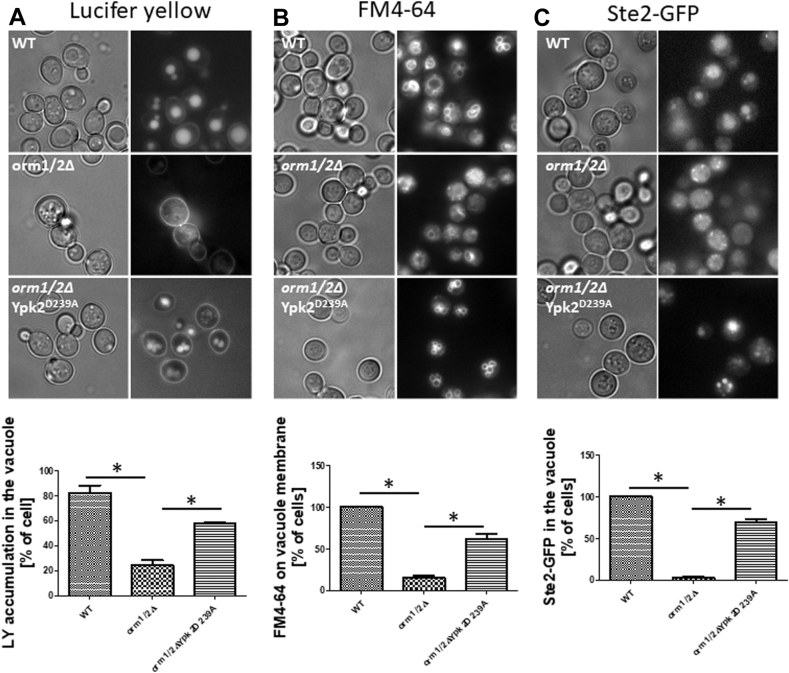
Defining an endocytosis defect of *orm1/2Δ*. Defective endocytosis of Lucifer yellow (A), FM4-64 (B), and Ste2-GFP (C) in *orm1/2Δ* yeast. Indicated yeast strains stained with Lucifer yellow, FM4-64 or containing GFP-tagged Ste2 were observed under a fluorescence microscope. These images are representative of three independent biological replicates. The graph beneath each figure shows the percentage of cells with normal accumulation of the indicated endocytic markers. Three randomly selected fields were counted blindly for each strain. ∗*P* < 0.05.

The above results revealed a surprising effect of Orm1/2 on CerS through the emerging regulatory module centered on Orms, LCBs, and Ypk1. Next, we sought to determine if Orm proteins use this module to control biological functions previously unappreciated as Orm-regulated. Endocytosis was tested first since Ypk1 is a major regulator of endocytosis through multiple mechanisms including regulating SPL production in yeast (61, 62, 63). To evaluate if Orm1/2 control yeast endocytosis and if this occurs through Ypk1, endocytosis in the *orm1/2Δ* strain was evaluated using specific cargo indicators. Uptake of the membrane-impermeant fluorescent compound Lucifer yellow (LY) into the lumen of the vacuole (the equivalent of mammalian lysosome) was used to measure fluid phase endocytosis (64). Instead of accumulating in the vacuole, a very faint LY signal was detected inside *orm1/2Δ* cells (Fig. 5A). Endocytosis of the plasma membrane was measured by using the lipid-soluble styryl dye FM4-64 (49). FM4-64 was transported from the plasma membrane to the limiting membrane of the vacuole in WT yeast. Accumulation of FM4-64 on the vacuole membrane was reduced greatly in *orm1/2Δ* yeast, with the appearance of punctate structures, presumably endosomes (Fig. 5B). Lastly, endocytosis of plasma membrane proteins was evaluated based on Ste2, a yeast mating pheromone α-factor receptor (65). In the absence of α-factor, plasma membrane Ste2-GFP undergoes constant endocytosis and accumulates in the vacuolar lumen (66). Instead of accumulating in the vacuole, Ste2-GFP appeared in punctate structures in *orm1/2Δ* yeast (Fig. 5C). In summary, all three assays indicated a clear defect in endocytosis in *orm1/2Δ* mutant yeast.

Mechanistically, the replacement of WT Ypk2 with constitutively active Ypk2^D239A^ almost completely reversed the endocytosis defects of *orm1/2Δ* (Fig. 5A–C). Taken together, these data reveal that Orm proteins control yeast endocytosis through the regulation of Ypk1.

### Defining an actin polarization defect of *orm1/2Δ*

**Fig. 6:**
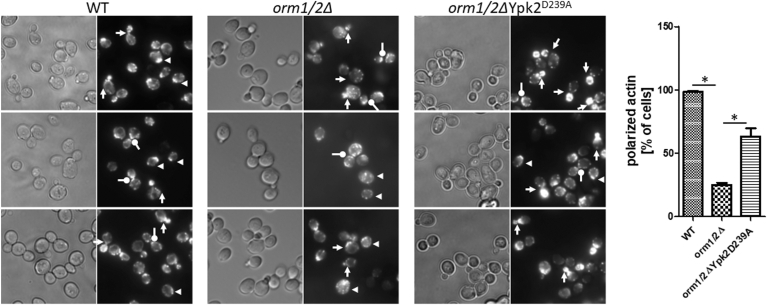
Actin polarization is defective in *orm1/2Δ* yeast. Indicated yeast were fixed and stained for actin with Rhodamine-phalloidin. Arrows of different forms point to cells at different stages of the cell cycle. Three fields of cells were randomly selected and visualized under both DIC and fluorescence optics. The graph on the right shows the percentage of cells with polarized actin patches.

Impaired endocytosis is often accompanied by an actin polarization defect, and Ypk1 is required for yeast actin polarization, that is, differential actin distribution between mother and daughter cells during the yeast cell cycle that supports the polarized growth of budding yeast (50, 55, 67). To test if Orm proteins also control actin polarization, rhodamine-phalloidin was used to label actin, and its distribution was observed via a fluorescence microscope (Fig. 6). WT yeast exhibited a normal distribution at various stages of the cell cycle. The actin cap was assembled at the pre-selected bud site on unbudded cells prior to S phase entry (arrowhead). For small- and middle-sized budded cells, actin patches were concentrated in the daughter cells and absent from mother cells (point-end arrow). Actin patches were relocated to the mother bud neck in large-budded cells prior to cytokinesis (round-end arrow). Cells lacking ORM1/2 exhibited a randomized distribution of actin patches between mother and daughter cells in all cell cycle stages in the majority of cells. This was most evident based on the appearance of large mother cells with intensive actin patch staining in *orm1/2Δ* yeast.

Importantly, introducing the Ypk2^D239A^ allele rescued the actin polarization defect in *orm1/2Δ*. These data demonstrate that Orm proteins are required for yeast actin polarization through the regulation of Ypk1.

## Discussion

**Fig. 7:**
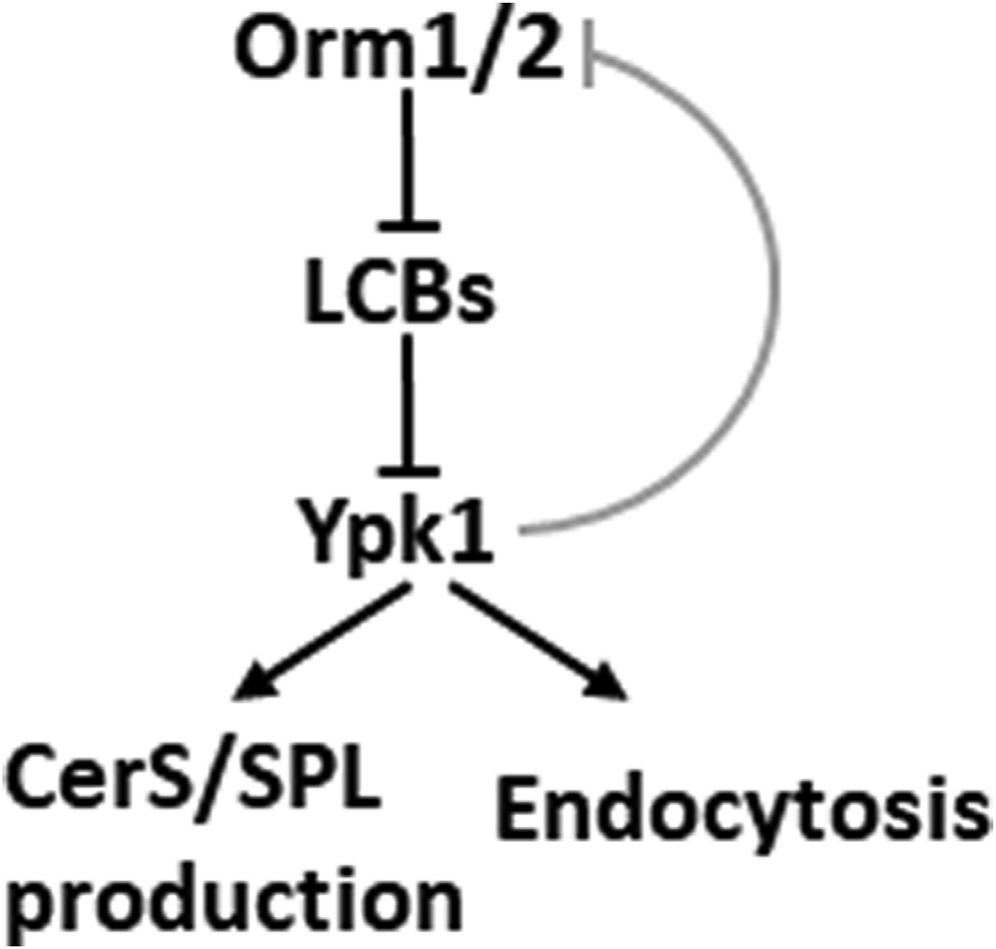
A model illustrating Orm1/2-LCB-Ypk1 signaling pathway. Orm proteins regulate LCB production to control Ypk1 and downstream SPL production and endocytosis. Phosphorylation of Orm1/2 by Ypk1 might serve as a feedback mechanism to reduce the activation signal.

In this study, we uncover important and complex regulation of SPL metabolism and function in yeast. We discover that LCB-induced Ypk1 inactivation leads to a defect in ceramide synthesis and to a major defect in endocytosis in *orm1/2Δ* mutants. The data support a novel model where Orm proteins functionally regulate LCB production to control Ypk1 and downstream effectors and biology, as shown here for CerS and endocytosis (Fig. 7). The model depicts the elucidation of important and novel functions of Orm proteins as major regulators of Ypk1 activity through controlling LCB production.

### The effects of Orm proteins on ceramide synthesis

Orm proteins have been established as major regulators of SPL production by controlling the activity of SPT. Yet it was noticed that there might be a possible connection between Orm proteins and ceramide synthesis. In yeast, decreased cellular ceramide level was reported in *orm1/2Δ*, suggesting the possible effect of Orm proteins in activating ceramide production (31, 68). Although our results do not show much difference in the steady-state level of ceramides in *orm1/2Δ*, we clearly demonstrate reduced in vivo CerS activity and a decrease in CerS activation at the molecular level demonstrated by reduced Lac1-phosphorylation. These results reveal the complexity and interconnectivity of the SPL biosynthetic pathway. It is noteworthy that in a mammalian system, where all three *ORMDL* genes were knocked out from A549 cells, a similar observation was made that LCBs accumulate at a much higher level than ceramides (dihydrosphingosine (33-fold) and dihydro-S1P (240-fold) versus dihydroceramides (6-fold)) (37). This suggests the effects of Orm proteins on ceramide synthesis might be a conserved mechanism beyond yeast.

### Role of Ypk1 in mediating the effects of Orm proteins on ceramide synthesis

Whereas one study suggested that Orm proteins may affect ceramide synthase activity through direct interaction with Lac1, our current results implicate Ypk1 as a critical mediator of the effects of Orm1/2 on Lac1/Lag1. YPK1 and its homolog YPK2 are essential genes, and they control various cellular processes through different substrates, including Lac1/Lag1. Yet it is unclear how Ypk1 activity is regulated in the cell except that it needs two upstream kinases, Pkh1/2 and Torc2, for activation. Future studies to investigate how Pkh1/2 or Torc2 activity are affected in *orm1/2Δ* will further elucidate the mechanism of this regulation. It is also known that Ypk1 phosphorylates Orm1/2 to release its inhibitory effect on SPT (60). This might serve as a feedback mechanism to reduce the activation signal. There is also a possibility that LCB regulators other than Orm1/2 control Ypk1/2 activity under certain physiological conditions.

### LCBs negatively regulate Ypk1 activity

Our study implicates Ypk1 as a key mediator downstream of LCBs, such that high elevation in LCB levels suppresses Ypk1 function. The connection between LCBs and Ypk1 was first proposed with LCBs as activators of Ypk1 by targeting and activating the upstream regulatory kinases Pkh1/2, a finding mainly based on an in vitro kinase assay (69, 70, 71). This was later questioned as LCB-induced Ypk1 phosphorylation was not observed in vivo (72). Then came the observation that phosphorylation of Ypk1 substrates are induced upon treating yeast with myriocin, a potent and specific inhibitor of SPT (29, 60). This suggested a role of SPLs in inhibiting Ypk1 activity, and complex SPLs, ceramides and LCBs have since all been suggested as the functional SPL species that regulate Ypk1 activity (73, 74, 75). In this study, we provide evidence that LCBs inhibit Ypk1 activation in *orm1/2Δ*: cellular LCB level is negatively correlated with Ypk1 activity; exogenously added LCBs induce rapid and transient Ypk1 inactivation; the inhibitory effect of exogenous LCBs on Ypk1 is not compromised in yeast mutants where the conversion of LCBs into downstream SPL products is compromised, thus ruling out a role for LCB-phosphates or ceramides. Most importantly, using the flux assay to monitor the incorporation of deuterated serine into the SPL pathway, we demonstrate that increased LCB production in *orm1/2Δ* is accompanied by decreased ceramide production. These results strongly argue that it is the LCBs, not the downstream SPL species, that exert a negative effect in their conversion into ceramides through inhibiting Ypk1. In order to establish the connection between LCBs and Ypk1, it is important to find the direct targets of LCBs. The potential SPL-binder Slm1 might serve as an LCB sensor because their translocation from eisosome to membrane compartment containing Torc2 (MCT) is regulated by LCB level and required for Ypk1 activation (73, 76, 77, 78, 79, 80, 81, 82). Since both sphingolipid metabolism and AGC family protein kinases are highly conserved between yeast and mammals, their interaction may represent a general signaling node.

### Endocytosis, a novel function of Orm proteins

Other than clarifying Orm proteins’ effects on SPL metabolism by demonstrating the connection between Orm and CerS, this study also reveals novel functions of Orm proteins in endocytosis and the actin cytoskeleton mediated by Ypk1. The role of Torc2/Ypk1 in endocytosis has long been demonstrated (61). Two mechanisms of function have been proposed, the rapid phosphorylation of phospholipid flippase kinases Fpk1/2 and the slower change in plasma membrane tension (62, 63, 83). Our study shows complicated changes in the SPL profile caused by deleting Orm genes, suggesting a possible role of SPL metabolism in endocytosis. In order to further understand Orm proteins’ function in endocytosis, it is important to study how Orm proteins are regulated under physiological conditions to promote endocytosis.

Taken together, in an attempt to characterize how Orm proteins affect SPL metabolism, we discovered key additional effects they have on SPL metabolism and a novel function in endocytosis through regulating Ypk1 signaling in an LCB-dependent manner. The study suggests an emerging Orm1/2-LCB-Ypk1 signaling module. Identifying the physiological conditions under which this module is utilized should increase our understanding of the biological function of Orm proteins.

## Data availability

All data are contained within the manuscript.

## Conflict of interest

The authors declare the following financial interests/personal relationships which may be considered as potential competing interests.

Dr Maurizio Del Poeta, MD, is a Co-Founder and Chief Scientific Officer (CSO) of MicroRid Technologies Inc. The goal of MicroRid Technologies Inc is to develop new anti-fungal agents of therapeutic use. All other authors declare no competing interests.
